# Dynamics of Fermentation Parameters and Bacterial Community in Rumen of Calves During Dietary Protein Oscillation

**DOI:** 10.3390/microorganisms12112123

**Published:** 2024-10-23

**Authors:** Kun Zhang, Zhanwei Teng, Qing Meng, Shuai Liu, Liping Yuan, Tong Fu, Ningning Zhang, Tengyun Gao

**Affiliations:** 1 College of Animal Science and Veterinary Medicine, Henan Institute of Science and Technology, Xinxiang 453003, China; 2 College of Animal Science and Technology, Henan Agricultural University, Zhengzhou 450046, China

**Keywords:** protein concentration oscillation, nitrogen utilization efficiency, fermentation parameters, plasma urea-N concentration, rumen bacteria

## Abstract

Dietary crude protein concentration oscillation can improve the nitrogen utilization efficiency of ruminants. However, little is known about the dynamic changes in microbiota and fermentation in the rumen of calves during the oscillation period. In this study, six calves were fed an oscillating diet at 2-day intervals, including a high-protein diet (HP) and a low-protein diet (LP). The rumen fermentation parameters, plasma urea-N concentration, and rumen bacterial diversity were characterized throughout the oscillation period. The concentrations of volatile fatty acids, NH_3_-N, and plasma urea-N in rumen changed significantly with an oscillating diet. The abundance of *Prevotella_1*, *Selenomonadales*, *Succiniclasticum*, *Clostridiales*, *Ruminococcaceae*, *Lachnospiraceae*, and *Rikenellaceae_RC9_gut*_group showed significant changes with diet. *Prevotella_1* was positively correlated, and *Lachnospiraceae_AC2044*_group and *Saccharofermentans* were negatively correlated with NH_3_-N. The abundance of Amino Acid Metabolism, Metabolism of Other Amino Acids, and Glycan Biosynthesis and Metabolism pathways, annotated by bacterial functional genes, decreased when the diet changed from HP to LP. The abundance of the Carbohydrate Metabolism pathway increased after the two dietary changes. In conclusion, the plasma urea-N concentration was not as sensitive and quick to adapt to diet changes as the rumen fermentation parameters. Rumen bacteria were responsible for increasing the nitrogen utilization efficiency of calves fed an oscillating diet.

## 1. Introduction

Cattle can digest plant resources and provide high quality food for human beings. However, the utilization efficiency of crude protein (CP) is low, which has a negative impact on the environment and increases feed costs [1]. A proposed method to improve nitrogen retention is to oscillate dietary CP concentration. Several studies have shown that dietary CP concentration oscillation (hereinafter referred to as an oscillating diet) at 2-day intervals can improve the nitrogen utilization efficiency of ruminants, thus reducing the amount of nitrogen needed to achieve optimal performance and reducing the release of nitrogen to environment [2,3,4]. Consistent with the observed differences in nitrogen retention, lambs fed an oscillating diet had higher body weight gain than those fed a static diet [4]. However, some studies found that the nitrogen retention of sheep did not improve when fed an oscillating diet [5]. This may be due to the fact that dietary nitrogen may have exceeded the requirements of these animals and rumen microorganisms, and the increase of nitrogen excretion masked the effect of the oscillating diet on nitrogen metabolism [3]. Although the effects of the oscillating diet on nitrogen retention are different in these studies, the performance of ruminants was rarely decreased when fed an oscillating diet compared with a static diet. Therefore, it may provide a potential way for cattle farmers to manage nutrient loss more closely in the future [6].

The rumen is the primary site for the microbial fermentation of ingested feed in ruminants [7]. With the degradation of feed substance in the rumen, a number of metabolites are produced and released, including volatile fatty acids (VFA) and nitrogenous compounds. These metabolites are either absorbed across the rumen epithelium or in the lower gastrointestinal tract and then enter the bloodstream for host use [8]. It is well known that diet can impact the structure of the rumen microbiome and may influence fermentation products. NH_3_-N is the main final product of dietary protein and non-protein nitrogen degradation and is the nitrogen form required by most bacteria in the rumen [8]. Therefore, rumen fermentation parameters can be used as an indicator of nitrogen metabolism in the rumen. Additionally, plasma urea-N concentration can also be assumed to give an indication of the availability of rumen nitrogen [9].

Dietary CP metabolized in the rumen is generally used for microbial synthesis. The nitrogen utilization efficiency of cattle may be affected by the activity of the rumen microorganisms that degrade feed CP. It is important to determine which microorganisms can decompose and utilize feed effectively according to the effects of different dietary protein concentrations on the rumen environment and microbial ecosystem. Bacteria are the most abundant microorganisms in the rumen of ruminants. Known bacteria involved in amino acid degradation include members of *Aminobacterium*, *Thermovirga*, *Aminivibrio* and *Clostridium hydroxybenzoicum* [10]. *Prevotella* is a very diverse genus, which can decompose both cellulose and protein [11,12,13]. Microbial symbiosis has inherent adaptability to nonsynchronous nitrogen and the energy supply of ruminants [14]. Studies have found that rumen protein degradation and NH_3_-N assimilation by rumen microbiota are not affected by an oscillating diet at 48 h intervals, which may be due to the adaptive ability of micro-organisms within 48 h or during malnutrition [15].

Dietary crude protein concentration oscillation may improve the nitrogen utilization efficiency of ruminants. Our previous studies have shown that oscillating diet at 2 day intervals improved the nitrogen utilization efficiency of calves compared with a static diet [16,17]. We hypothesized that crude protein concentration oscillation improved nitrogen utilization efficiency by affecting rumen microbiota. In this study, the rumen fermentation parameters, NH_3_-N, plasma urea-N concentration, and rumen bacterial diversity were characterized throughout the oscillation period to elucidate the mechanism of crude protein concentration oscillation enhancing nitrogen utilization efficiency, thereby providing a foundation for reducing nitrogen emissions and feed costs in dairy cow production.

## 2. Materials and Methods

### 2.1. Animal Experiment

This study was carried out on the basis of an agreement approved by the Theoretic Committee on Animal Protection and the use of Henan Institute of Science and Technology (approval No. LLSC2023023), and all procedures involving calves were in compliance with the guidelines of the Protocol of Animal Welfare for Bovine During Breeding, Transport, and Slaughter (SN/T 3774-2014). Six 4-month-old Holstein calves with similar body weights (about 94.3 kg/head) were housed in individual hutches at Henan Agricultural University, Practical and Teaching Base. The calves were fed an oscillating diet at 2-day intervals, including a high-protein diet (HP, 173 g/kg) and a low-protein diet (LP, 125 g/kg). The nutritional composition of the two diets was as consistent as possible, except for the crude protein level. The average protein level of the diets within four days was 149 g/kg, which supported moderate body weight gain for calves [18]. The ingredients and nutrient level of the basal diets are shown in Table 1. The calves were fed twice daily at 08:00 h and 17:00 h. In order to characterize the rumen microorganisms changing with dietary protein level, the calves had equal daily dry matter feeding. The experiment lasted for 24 days, including 20 days (four oscillation periods) of adaptation and 4 days (one oscillation period) of sampling.

### 2.2. Sample Collection

During the 4-day sampling period, the rumen fluid samples were collected using an oral stomach tube connected to a vacuum pump through the esophagus [19] 2 h after morning feeding on days 21, 22, 23 and 24, and labeled as HP1 (the first day of HP), HP2 (the second day of HP), LP1 (the first day of LP), and LP2 (the second day of LP), respectively. The collected samples were immediately measured with a portable pH meter and filtered through sterile gauze for the determination of acetic acid, propionic acid, butyric acid, and NH_3_-N concentrations, and some of the samples were immediately frozen in liquid nitrogen for further DNA extraction. Blood samples were obtained from the jugular vein after the calves were fasted for 8 h before morning feeding on days 22, 23, 24 and 25 and were labeled as HP1, HP2, LP1 and LP2, respectively, and then centrifuged at 2000× *g* at room temperature for 15 min to separate plasma.

### 2.3. Plasma Urea-N, NH_3_-N, and VFA

10 mL of rumen fluid was mixed with 1 mL of 10% (*w*/*v*) sulfuric acid and centrifuged (at 4300× *g* for 20 min and then 12,000× *g* for 20 min, 4 °C) [20]. The supernatant of 1 mL was used to determine the concentration of NH_3_-N according to the method described by Broderick and Kang [21]. Another 1 mL of this supernatant was filtered through a 0.22-micron membrane to determine the VFA with ion chromatography. Samples were injected with an autosampler (S5300; Sykam GmbH, Munich, Germany) into a Dionex AS11-HC column 4 × 250 mm (Thermo Fisher Scientific, Waltham, MA, USA) on an S150 Sykam ion chromatography system. Chromatographic separation was carried out using a solvent system consisting of two eluents, a 0.5 mmol/L NaOH held for 25 min, then 50 mmol/L NaOH for 5 min, and finally 0.5 mmol/L NaOH for 10 min, with all samples analyzed in duplicate. The plasma urea-N concentration was detected by diacetyl oxime colorimetry using a urea assay kit (Nanjing Jiancheng Bioengineering Institute, Nanjing, China).

### 2.4. DNA Extraction, 16S rRNA Gene Amplification, and Sequencing

Total DNA was extracted from the rumen fluid using a Bacterial DNA Kit (Omega), its concentration was measured using a NanoDrop 1000 Spectrophotometer (NanoDrop Technologies Inc., Wilmington, DE, USA), and DNA quality was checked using 1% agarose gel electrophoresis. The V3 and V4 regions of the 16S rRNA gene were amplified by PCR using the universal primers 338F (5′-ACTCCTACGGGAGGCAGCAG-3′) and 806R (5′-GGACTACHVGGGTWTCTAAT-3′) [22]. The amplified products were purified, quantified, and homogenized to prepare the amplified library. All libraries were sequenced using an Illumina HiSeq 2500 (Illumina, San Diego, CA, USA) at Biomarker Technologies Co, Ltd. (Beijing, China). The 16S rRNA sequence data sets are publicly available through NCBI’s Sequence Read Archive, under accession number PRJNA722793.

### 2.5. Processing of Sequencing Data

The original image data files obtained by sequencing were transformed into sequenced reads with a base calling analysis. Raw tags were obtained by overlapping the stitching reads of each sample using FLASH, filtered with Trimmomatic, and chimeras were removed with UCHIME v4.2 software to obtain effective tag sequences. The effective tags were clustered into operational taxonomic units (OTUs) at a 97% similarity level and filtered at 0.005% of the sequence number as a threshold. The alpha-diversity index (including the Chao1, Ace, Shannon, and Simpson indexes) was evaluated using Mothur. Beta-diversity was analyzed using QIIME, and the distance between the samples was calculated by weighted UniFrac. OTUs were annotated based on the Silva (bacteria) taxonomic database (Release 128, http://www.arbsilva.de, accessed on 14 November 2019). The discriminative bacteria that most likely explain differences between groups were determined by LEfSe (Line Discriminant Analysis (LDA) Effect Size). The Reconstruction of Unobserved States (PICRUSt) analysis was used for predicting the differential functional genes of bacterial communities and annotated in the KEGG pathway.

### 2.6. Statistical Analysis

The differences of rumen fermentation parameters (i.e., pH, concentration of NH_3_-N, acetic acid, propionic acid, and butyric acid) and plasma urea-N between groups were analyzed with one-way analysis of variance (ANOVA) followed by Duncan’s multiple comparison test using SPSS Statistics 22 (IBM., Armonk, NY, USA). Pearson’s correlation analysis between bacteria (at the phylum level and the top 30 genera in abundance) and fermentation parameters in rumen were carried out, and the thermogram was drawn using BMKCloud (www.biocloud.net, accessed on 20 July 2020). The significance threshold for all statistical analyses was set to *p* < 0.05.

## 3. Results

### 3.1. Rumen Fermentation Parameters and Plasma Urea-N Concentration Changed with an Oscillating Diet

**Figure 1:**
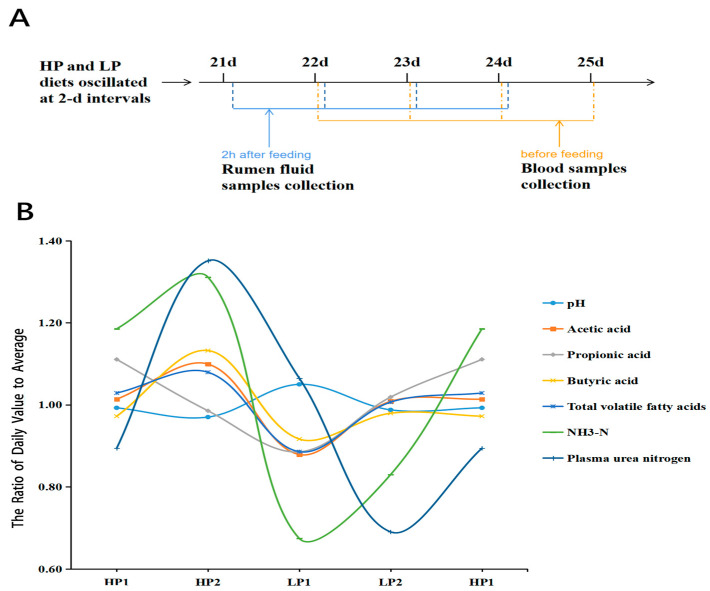
Rumen fermentation and plasma urea-N of calves are susceptible to an oscillating diet. (**A**) experimental design. (**B**) the variation curve of Rumen fermentation parameters and plasma urea-N concentration after the 4 days of one oscillation period. HP1, the first day of the high-protein diet for calves; HP2, the second day of the high-protein diet for calves; LP1, the first day of the low-protein diet for calves; LP2, the second day of the low-protein diet for calves.

Rumen fluid and blood samples were collected for four consecutive days to characterize the dynamic changes in fermentation parameters and plasma urea-N concentration with an oscillating diet (Figure 1A). As shown in Table 2, the pH value of rumen fluid in LP1 was significantly higher than HP1, HP2 and LP2, which was opposite to the concentration of total VFA. The rumen acetic acid concentration in HP2 was significantly higher than HP1, LP1, and LP2, and in HP1 and LP2 it was significantly higher than in LP1. The rumen propionic acid concentration in HP1 was significantly higher than in LP1, but there was no significant difference before and after the two dietary changes. The concentration of butyric acid in rumen did not change significantly during the oscillation period. The rumen The NH_3_-N concentration in HP2 was significantly higher than in LP1 and LP2, and in HP1 it was significantly higher than in LP1. The plasma urea-N concentration in HP2 was significantly higher than HP1, LP1 and LP2, and in LP1 it was significantly higher than in LP2.

In addition, we showed the variation curve of these parameters during the oscillation period, which was expressed by the ratio of the daily value to the average within four days to eliminate the large differences of absolute values between the parameters (Figure 1B). The pH value increased when the diet changed from HP to LP, and recovered in LP2. The propionic acid concentration was affected on the first day of dietary changes and recovered on the second day of the same diet. The concentrations of acetic acid, total VFA, and NH_3_-N decreased when the diet changed from HP to LP and recovered in LP2, increased from LP to HP, and reached their highest levels in HP2. The plasma urea-N concentration decreased when the diet changed from HP to LP, reaching its highest levels in HP2 and lowest levels in LP2.

### 3.2. Oscillating Diet Altered the Diversity of the Rumen Microflora in Calves

**Figure 2:**
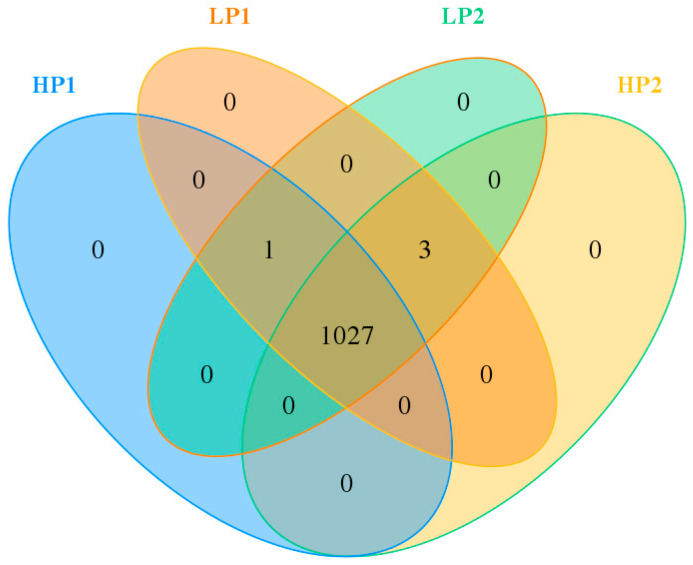
Venn diagram of core OTUs in the rumen of calves in the 4 days of one oscillation period. HP1, the first day of the high-protein diet for calves; HP2, the second day of the high-protein diet for calves; LP1, the first day of the low-protein diet for calves; LP2, the second day of the low-protein diet for calves.

Bacterial communities from 24 rumen fluid samples were evaluated by sequencing the V3 and V4 regions of the 16S rRNA gene, and an average of 75,817 effective sequences were obtained from each sample. As shown in Figure 2, a total of 1031 OUTs were identified in all samples, among which 1027 OTUs were found across the 4 days of one oscillation period and accounted for 99.6% of the total OTUs, indicating a high shared richness. OTU477, which was classified into the genus *Comamonas*, did not exist in HP2 samples. OTU32, OTU221, and OTU547, which were classified into the genera *Syntrophococcus*, *[Eubacterium]_oxidoreducens*_group, and uncultured_bacterium_o_*WCHB1-41*, respectively, did not exist in HP1 samples.

**Figure 3:**
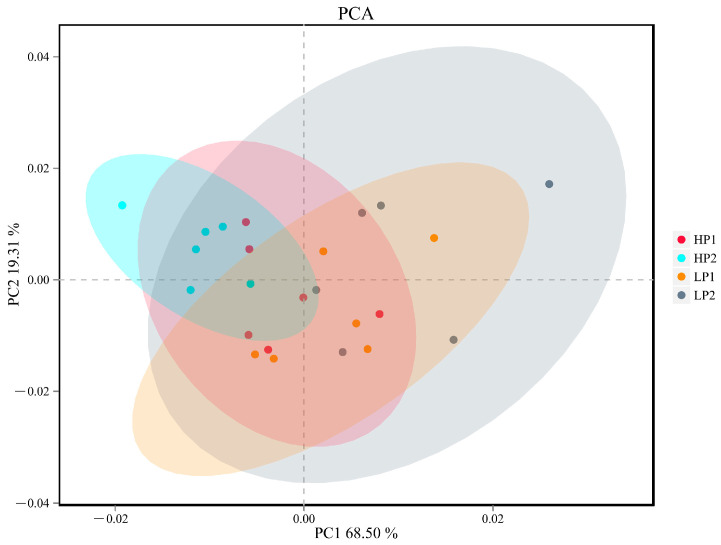
The principal component analysis (PCA) of the OTUs in the rumen of calves in the 4 days of one oscillation period. PC1 and PC2 explained 68.50% and 19.31% of variation, respectively. HP1, the first day of the high-protein diet for calves; HP2, the second day of the high-protein diet for calves; LP1, the first day of the low-protein diet for calves; LP2, the second day of the low-protein diet for calves.

According to the results of alpha-diversity analysis (Table 3), there was no significant difference in the richness and diversity of rumen bacterial communities within 4 days. To measure the extent of the distinction between microbial communities, PCA was performed. There was no obvious cluster in any of the four days (Figure 3).

### 3.3. An Oscillating Diet Disturbed the Microflora Composition in the Rumen of Calves

**Figure 4:**
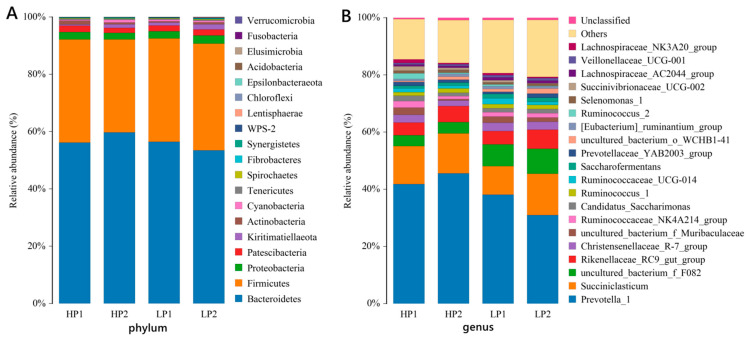
The relative abundance of bacteria in the rumen of calves in the 4 days of one oscillation period. Relative abundances at the (**A**) phylum and (**B**) genus levels for bacteria that exceeded 1% of the total.

OTUs were annotated with the corresponding bacteria classification information at each level (phylum, class, order, family, genus) based on the Silva (bacteria) taxonomic database. In the analysis of rumen bacterial community composition, the relative abundance at the level of the two most representative phyla and genera were selected. A total of 19 phyla were annotated (Figure 4A). There were six phyla with relative abundances higher than 1%, among which *Bacteroidetes*, *Firmicute*, *Proteobacteria*, and *Patescibacteria* were shared across the 4 days. The abundance of *Kiritimatiellaeota* was higher than 1% in HP2, LP1 and LP2, and *Actinobacteria* was only higher than 1% in HP1. A total of 158 genera were annotated, including 154, 153, 156, and 157 genera in HP1, HP2, LP1, and LP2, respectively. As shown in Figure 4B, there were 13 genera with an abundance greater than 1%, and the most abundant genera were *Prevotella_1*, *Succiniclasticum*, uncultured_bacterium_f_*F082*, *Rikenellaceae_RC9*_gut_group, and *Christensenellaceae_R-7*_group.

**Figure 5:**
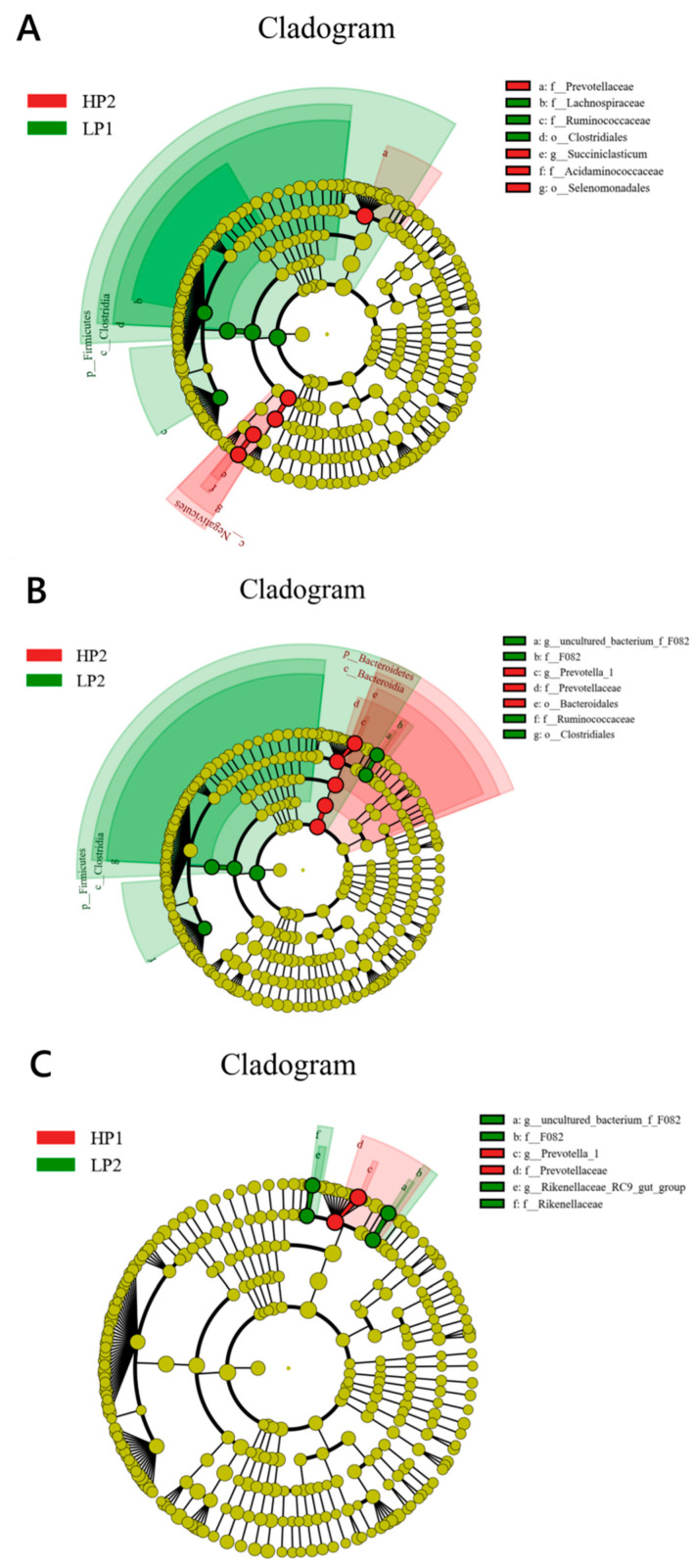
The bacteria with statistical differences in the rumen of calves between (**A**) HP2 and LP1, (**B**) HP2 and LP2, and (**C**) LP2 and HP1, found by LEfSe. The circles radiating from inside to outside represent the taxonomic level from phylum to genus; each small circle represents a taxonomy under this level, whose diameter is proportional to its relative abundance; the yellow node represents the microflora that do not play an important role in different groups, and other different microflora are colored according to the group with a higher abundance. HP1, the first day of the high-protein diet for calves; HP2, the second day of the high-protein diet for calves; LP1, the first day of the low-protein diet for calves; LP2, the second day of the low-protein diet for calves.

The biomarkers with statistical differences between groups were found by LEfSe. There were seven different taxa between LP1 and HP2. The abundance of *Clostridiales* (order), *Lachnospiraceae* (family), and *Ruminococcaceae* (family) was higher, while the abundance of *Selenomonadales* (order), *Prevotellaceae* (family), *Acidaminococcaceae* (family), and *Succiniclasticum* (genus) was lower in LP1 than in HP2 (Figure 5A). There were seven different taxa between LP2 and HP2. The abundance of *Clostridiales* (order), *Ruminococcaceae* (family), *F082* (family), and uncultured_bacterium_f_*F082* (genus) was higher, while the abundance of *Bacteroidales* (order), *Prevotellaceae* (family), and *Prevotella_1* (genus) was lower in LP2 than in HP2 (Figure 5B). There were six different taxa between HP1 and LP2. The abundance of *Prevotellaceae* (family) and *Prevotella_1* (genus) was higher, while the abundance of *F082* (family), *Rikenellaceae* (family), uncultured_bacterium_f_*F082* (genus), and *Rikenellaceae_RC9*_gut_group (genus) was lower in HP1 than in LP2 (Figure 5C).

**Figure 6:**
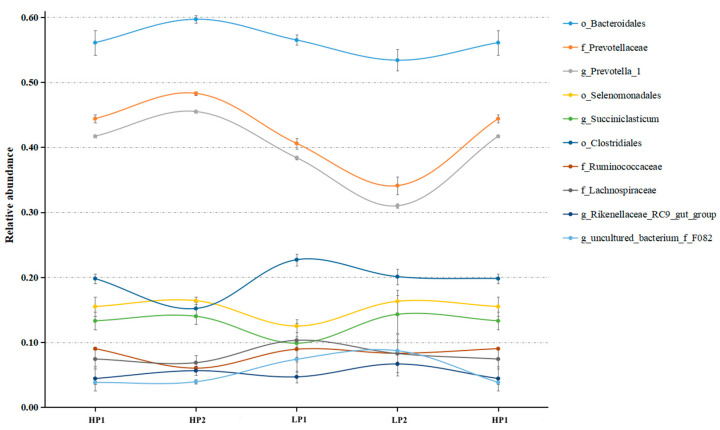
The variation curves of differential bacteria in the 4 days of one oscillation period. HP1, the first day of the high-protein diet for calves; HP2, the second day of the high-protein diet for calves; LP1, the first day of the low-protein diet for calves; LP2, the second day of the low-protein diet for calves.

The variation curves of these differential bacteria within 4 days are shown in Figure 6. The abundance of *Bacteroidales*, *Prevotellaceae*, and *Prevotella_1* increased in HP and was highest in HP2, decreasing in LP and reaching its lowest level in LP2. The abundance of the genus uncultured_bacterium_f_*F082* (equivalent to the family *F082*) increased from HP to LP, and reached its highest level in LP2. The changes in these bacteria may be influenced by dietary protein concentrations. The abundances of *Selenomonadales*, *Succiniclasticum*, *Clostridiales*, *Ruminococcaceae*, *Lachnospiraceae*, and *Rikenellaceae_RC9*_gut_group showed significant changes with dietary protein levels, and recovered on the second day of the same diet, indicating that a time-dependent effect on some bacteria before stabilization may have occurred as the days progressed.

### 3.4. An Oscillating Diet Altered the Microbiota-Associated Functions

**Figure 7:**
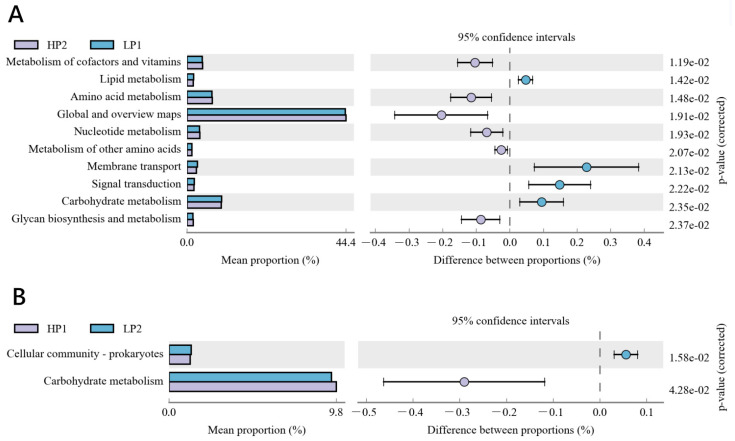
The differential KEGG pathway predictions of the bacterial communities between (**A**) HP2 and LP1, and (**B**) LP2 and HP1. The figure shows the relative proportion and differences of KEGG pathways within 95% confidence intervals, and the rightmost value is the *p* value (<0.05). HP1, the first day of the high-protein diet for calves; HP2, the second day of the high-protein diet for calves; LP1, the first day of the low-protein diet for calves; LP2, the second day of the low-protein diet for calves.

The relative abundance of KEGG metabolic pathways annotated by functional genes of rumen bacteria was inferred by PICRUSt. Figure 7 shows the KEGG pathway with a relative abundance of greater than 1%. There were 10 KEGG pathways that showed significant differences when the diet changed from HP to LP (Figure 7A), among which the relative abundances of Metabolism of Cofactors and Vitamins, Amino Acid Metabolism, Nucleotide Metabolism, Metabolism of Other Amino Acids, and Glycan Biosynthesis and Metabolism decreased. It can be inferred that the abundance of rumen bacteria involved in amino acid, nucleotide, and glycan metabolism decreased when dietary protein concentration changed from high to low. The relative abundances of Lipid Metabolism, Membrane Transport, Signal Transduction, and Carbohydrate Metabolism increased when the diet changed from HP to LP. The relative abundances of Cellular Community-Prokaryotes decreased and Carbohydrate Metabolism increased when the diet changed from LP to HP (Figure 7B). Due to this, the relative abundance of Carbohydrate Metabolism pathway increased after the two dietary changes.

### 3.5. The Relationship Between the Rumen Bacteria and Fermentation Parameters

**Figure 8:**
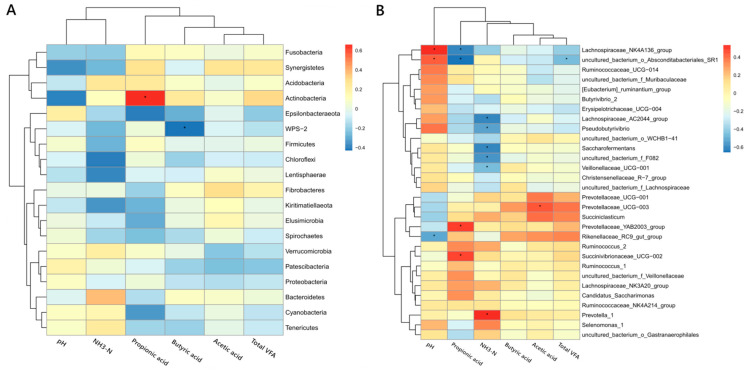
Thermogram of the correlation between bacteria and fermentation parameters in the rumen and plasma urea-N of calves, * indicated significance. At (**A**) the phylum level and (**B**) the top 30 genera in abundance for bacteria. Rumen fermentation parameters included pH, and concentrations of acetic acid, propionic acid, butyric acid, total VFA and NH_3_-N. The total VFA is the sum of acetic acid, propionic acid, and butyric acid.

Pearson’s correlation analysis was performed between the bacteria and fermentation parameters. At the phyla level (Figure 8A), *Actinobacteria* was positively correlated with propionic acid. *WPS-2* was negatively correlated with butyric acid. At the genera level (Figure 8B), *Lachnospiraceae_NK4A136*_group was positively correlated with pH, and negatively correlated with propionic acid. uncultured_bacterium_o_*Absconditabacteriales_SR1* was positively correlated with pH, and negatively correlated with propionic acid and total VFA. *Lachnospiraceae_AC2044*_group, *Pseudobutyrivibrio*, *Saccharofermentans*, uncultured_bacterium_f_*F082*, and *Veillonellaceae_UCG-001* were negatively correlated with NH_3_-N. *Prevotellaceae_UCG-003* was positively correlated with acetic acid. *Prevotellaceae_YAB2003*_group and *Succinivibrionaceae_UCG-002* were positively correlated with propionic acid. *Rikenellaceae_RC9*_gut_group was negatively correlated with pH. *Prevotella_1* was positively correlated with NH_3_-N.

## 4. Discussion

Several studies have demonstrated that feeding with an oscillating diet at 48 h intervals can enhance the nitrogen retention in lambs [3,4] and steers [2] relative to those consuming a similar amount of CP at a static concentration. Our study found that oscillating diet also improved the nitrogen utilization efficiency of calves [16,17]. It is well known that the nutrient utilization efficiency of ruminants is closely related to rumen fermentation and influenced by diet. Our study showed significant changes in rumen fermentation parameters with dietary protein levels, including pH, acetic acid, propionic acid, total VFA, and NH_3_-N. However, these parameters only changed on the first day of LP and recovered on the second day. This may be due to the fact that a change in diet changes the rumen fermentation and the composition of microbial communities in a short time, but then it will return to the original state after a certain time due to the ability of microflora to resist stimulation and maintain stability [23]. In the two diets in this study, the carbohydrate forms were slightly different, while the gross energy was equal, so it is understandable that the rumen pH and VFA concentrations changed with the diet and recovered on the second day of the same diet, which indicated that the energy fermentation (or metabolism) mode in the rumen could adapt to the carbohydrate composition of different diets over a period of time.

NH_3_ is the final product of protein degradation in the rumen. It is fully absorbed and utilized by microorganisms, after which the concentration of surplus NH_3_-N in the rumen fluid begins to increase [24]. In this study, the NH_3_-N concentration in the rumen of calves increased when the dietary protein level changed from low to high, which was consistent with the result that rumen NH_3_-N concentration tended to be higher in ruminants fed a high-CP diet compared with those fed a low-CP diet [25,26]. The efficiency of nitrogen capture is important for the creation of readily available and utilizable sources of energy for microbial protein synthesis. The increased concentration of NH_3_-N is suggestive of an increased rate of proteolysis and amino acid metabolism in animals [27]. In addition, the plasma urea-N concentration can be considered an indicator of rumen nitrogen availability [9]. In this study, the plasma urea-N concentration changed with the dietary CP concentration, reaching its highest level in HP2 and its lowest in LP2, which supported the above view. The rumen NH_3_-N concentration and plasma urea-N concentration changed significantly with the oscillating diet. On the second day of LP, the reduced rumen NH_3_-N concentration slightly increased, while the plasma urea-N concentration continued to decrease. This demonstrated that the plasma urea-N concentration was not as sensitive and quick to adapt to the changes in diet as the rumen fermentation parameters.

Rumen microorganisms play an important role in the degradation of dietary nutrients. Diet can affect the overall alpha- and beta-diversity of rumen microbiota, and microbial diversity may affect feed efficiency in ruminants as well [8]. However, the data generated from this study would suggest the oscillating diet does not create imbalances in the rumen microbiota, as there was no significant difference in microbial alpha-diversity among the four days. However, PCA could separate HP2 from LP2, indicating that the composition of microbiota changed with the change of diet and time.

*Bacteroides* and *Firmicutes* are usually dominant in the rumen bacteria of cattle [27,28], and the same was true of calves in this study. It has been reported that the members of *Bacteroidetes* are the major rumen microorganisms that degrade non-structural carbohydrates and non-fibrous polysaccharides, while *Firmicutes* are the major rumen microorganisms that degrade structural carbohydrates [29,30].

*Prevotellaceae,* polysaccharide-degrading bacteria belonging to *Bacteroidetes* phyla, was the dominant family in the rumen of calves, which was consistent with previous studies in steers and cows [28,31]. Studies have shown that the relative abundances of *Prevotellaceae* [32], and the genera *Prevotella_1*, *Prevotellaceae UCG-001*, and *norank f Bacteroidales UCG-001* [28] are positively correlated with the concentration of propionic acid in rumen. Similarly, *Prevotellaceae_YAB2003*_group and *Succinivibrionaceae_UCG-002* were positively correlated with the concentration of propionic acid in this study. In addition, the members of *Prevotellacea* are also involved in the assimilation of NH_3_ within the rumen and microbial protein synthesis [33]. The relative abundance of *Prevotellacea* decreased when the dietary protein level changed from high to low, and vice versa, which was consistent with the changes in NH_3_-N concentration, indicating that the protein-degrading bacteria no longer belonged to the dominant flora at a low dietary protein concentration. The genus *Prevotella_1* is reported to be the most prevalent bacterial functional group, predominant in the rumen microbiome consortium [28]. *Prevotella_1* has the capability to degrade starches, simple sugarsm and other non-cellulosic polysaccharides as energy substrates to produce succinate, acetate, butyrate, and propionate [34,35]. In this study, no correlation between *Prevotella_1* and VFA was found, which may be due to the multiple niches of *Prevotella_1* in the rumen. It has been reported that a low concentration of NH_3_-N may result from the decreased protein degradation affected by the relative abundance of *Prevotella_1* in the rumen of steers [28]. Direct feeding with the members of *Prevotella* to cows significantly enhanced rumen NH_3_-N concentrations [36]. The relative abundances of the genera *Prevotellaceae_UCG-001* and *Prevotella_1* has been positively correlated with the NH_3_-N concentration in the rumen [28,37]. This was consistent with our finding of positive correlations between the abundance of *Prevotella_1* and NH_3_-N concentrations, which supports the view that *Prevotella_1* plays a role in the degradation of protein and peptides [34].

*Rikenellaceae* is a dominant family and involved in the degradation of structural carbohydrates and starch in the rumen of cows [28,38,39]. *Rikenellaceae_RC9*_gut_group was a genus with high abundance in this family, which was consistent with previous studies [28,40]. In this study, the abundance of *Rikenellaceae_RC9*_gut_group was negatively correlated with rumen pH, which was consistent with previous findings in the rumen of steers; that is, the abundance of *Rikenellaceae_RC9*_gut_group was positively correlated with the total VFA concentration and the molar proportion of acetate [28]. Inconsistent with the above results, the concentrations of total VFA, acetate, and propionate were negatively correlated with *Rikenellaceae_RC9*_gut_group abundances in the rumen of yak [37]. Therefore, we speculate that the abundance of this genus may also be affected by other factors, such as the negative correlation between its abundance and rumen NH_3_-N concentration [37].

Uncultured_bacterium_f_*F082*, belonging to *Bacteroidetes* phylum, was the dominant genus and negatively correlated with rumen NH_3_-N concentration. The abundance of the *F082* family increased with the change in dietary protein level from high to low, and reached the highest level in LP2, indicating that *F082* could adapt to the rumen fermentation conditions and gradually become the dominant bacteria with the adaption time of LP. However, information is limited on the ecology and biology of this taxa. Further studies to identify the function of members within *F082* are required to determine whether this taxon is linked to nitrogen metabolism in the rumen.

Members of the order *Clostridiales* (belonging to the *Firmicutes* phylum) have been found to be the dominant lignocellulolytic and polysaccharolytic bacteria that contribute to the production of VFA in the rumen [35,41]. The family *Lachnospiraceae* and *Ruminococcaceae* belonging to this order were found to be the dominant family in the rumen of steers [28] and to play important roles in the degradation of cellulose and fermentation of plant fibers [42]. Previous studies have shown that *Lachnospiraceae* was positively correlated with some endoglucanases, and enhanced carboxymethyl cellulose degradation during treatment with rumen microorganisms [43,44]. In this study, the abundance of *Clostridiales*, *Ruminococcaceae,* and *Lachnospiraceae* increased significantly when the dietary protein concentration changed from high to low, and recovered on the second day of LP, which supports the idea that subtle differences in the composition of cellulolytic bacteria may occur as the days advance but may reach a relatively balanced population for fiber degradation [45]. However, in this study, the genus *Lachnospiraceae_NK4A136*_group, a member of the *Lachnospiraceae* family, was positively correlated with pH and negatively correlated with propionic acid. Therefore, members of this family may also be regulated by other factors. In fact, negative correlations were found between nitrogen retention and *Butyrivibrio* and *Lachnospiraceae* in beef cattle [46], and we also found negative correlations between NH_3_-N concentration and *Lachnospiraceae_AC2044*_group and *Pseudobutyrivibrio* in the rumen of calves. For members of the family *Ruminococcaceae*, the abundances of *Ruminococcus_2* [28], *Ruminococcaceae_UCG-005*, *Ruminococcaceae_UCG-010*, and *Ruminococcaceae_NK4A214*_group [37] were negatively correlated with NH_3_-N concentration in the rumen, and we also found that there was a negative correlation between *Saccharofermentans* and NH_3_-N concentration. Therefore, *Lachnospiraceae* and *Ruminococcaceae* may be directly involved in nitrogen metabolism or indirectly affect microbial protein synthesis by participating in cellulose degradation and plant fiber fermentation in the rumen, based on the fact that the rumen microbiota gain energy from glucose units derived from the fiber degradation used to drive microbial protein synthesis and proliferation [33].

The genus *Succeniclasticum*, belonging to *Firmicutes* phylum, was the dominant bacteria in the rumen and was time-dependent on the oscillating diet of the calves, which may be to adapt to the changes in dietary carbohydrate composition [47]. *Succiniclasticum* is a propionate-producing taxon and is known to produce propionate through succinate decarboxylation [35]. The relative abundance of *Succiniclasticum* was also closely correlated with acetate and valeric acid population [28,37]. However, no correlation between VFA and this genus was found in this study. We are unable to explain this based on current research results.

Another interesting observation was the relative abundances of *Kiritimatiellaeota* were higher than 1% in HP2, LP1, and LP2 and lower than 1% in HP1, which indicated a sudden decrease when dietary protein level changed from low to high. Similarly, the decrease in lignocellulose and increase in the nitrogen content of the diet changed the rumen microbiota structure, reducing the relative abundances of *Kiritimatiellaeota*, which have been identified as fiber degraders [48,49]. However, they recovered to more than 1% on the second day of HP. We speculated that *Kiritimatiellaeota* may be sensitive to changes in diet.

*Actinobacteria* has been positively correlated with NH_3_-N in the rumen of lambs [50]. Although this correlation was not found in this study, the abundance of *Actinobacteria* was higher than 1% only in HP1. Therefore, *Actinobacteria* may be related to rumen metabolism of dietary protein and be time-dependent.

The change in rumen microbiota with an oscillating diet is ultimately reflected in its function. There were 10 KEGG pathways with a significant difference between LP1 and HP2 and enriched by functional bacteria. The abundance of functional bacteria involved in Amino Acid Metabolism, Nucleoside Metabolism, and Glycan Biosynthesis and Metabolism in the rumen decreased when the dietary protein level changed from high to low, which was consistent with the rumen NH_3_-N concentration, total VFA concentration, and plasma urea-N concentration. These changes in functional bacteria may be the reason for the increase of nitrogen utilization efficiency in calves fed an oscillating diet. The biological transformation of nitrogen molecules depends on sufficient energy support, so protein and energy metabolism are often synchronized in ruminants [51]. Studies have suggested that protozoa and bacteria can synthesize and store polysaccharides during a nitrogen deficiency period, and these polysaccharides can be used with sufficient nitrogen supply later [6]. Therefore, it is reasonable that the relative abundance of carbohydrate metabolism in functional bacteria increased, regardless of whether dietary protein level changed from high to low or from low to high. It may also be due to the fact that calves increase energy consumption in response to the stress of a diet change by increasing the microbial community involved in energy metabolism [52].

## 5. Conclusions

In this study, the concentrations of VFA, NH_3_-N in the rumen, plasma urea-N concentration, and rumen bacterial diversity were characterized during a dietary protein oscillation period. Protein oscillation affected fermentation parameters and NH_3_-N, which are associated with a change in bacterial diversity. The rumen bacteria may be more effective in utilizing dietary protein and recycled nitrogen during the oscillation period, which is responsible for increasing the nitrogen utilization efficiency of calves.

## Figures and Tables

**Table 1:** Ingredients and nutrient composition of diets.

Items	High-Protein Diet	Low-Protein Diet
Ingredient, g/kg of dry matter		
Corn, ground	337	420
Soybean meal	195	126
Corn protein powder	21.6	0
Wheat bran	63	71.5
Soybean oil	2.4	0
Limestone	12	11
Dicalcium phosphate	6	8.5
Salt	6.5	6.5
Vitamin–mineral premix ^1^	6.5	6.5
Oat grass, chopped	350	350
Total	1000	1000
Chemical composition, g/kg of dry matter (unless noted)		
Crude protein	173	125
Fat	30.7	44.5
Neutral detergent fiber	342	344
Acid detergent fiber	117	120
Crude ash	47.2	44.3
Calcium	8.7	8.7
Phosphorus	4.8	4.8
Net energy for maintenance (Mcal/kg) ^2^	1.89	1.9
Net energy for gain (Mcal/kg) ^2^	1.25	1.25
Gross Energy (Mcal/kg)	4.38	4.33

^1^ Containing per kilogram of supplement: 15,000 IU of vitamin A, 5000 IU of vitamin D, 50 mg of vitamin E, 90 mg of Fe, 12.5 mg of Cu, 60 mg of Mn, 100 mg of Zn, 0.3 mg of Se, 1.0 mg of I, and 0.5 mg of Co. ^2^ Calculated from tables of feed composition and nutritive values in China (2018 28th Edition; database, 2018).

**Table 2:** Rumen fermentation parameters and plasma urea-N concentration after the 4 days of one oscillation period.

Items	HP1	HP2	LP1	LP2	SEM	*p* Value
pH	6.4 ^b^	6.2 ^b^	6.7 ^a^	6.3 ^b^	0.05	<0.001
Acetic acid (mg/L)	4042.2 ^b^	4383.3 ^a^	3504.7 ^c^	4021.2 ^b^	82.9	<0.001
Propionic acid (mg/L)	1502.6 ^a^	1333.1 ^ab^	1197.8 ^b^	1378.2 ^ab^	35.8	0.014
Butyric acid (mg/L)	875.9	1020.1	825.8	882.4	28.9	0.092
Total volatile fatty acids (mg/L)	6420.7 ^a^	6736.5 ^a^	5528.3 ^b^	6281.9 ^a^	121.4	<0.001
NH_3_-N (mg/dL)	27.8 ^ab^	30.8 ^a^	15.8 ^c^	19.5 ^bc^	1.9	0.005
Plasma urea-N (mmol/L)	3.8 ^bc^	5.7 ^a^	4.5 ^b^	2.9 ^c^	0.3	<0.001

*n* = 6 for each treatment. *p* value < 0.05 was defined as statistically significant. Different letters within a row represent significant differences (*p* < 0.05). HP1, the first day of the high-protein diet for calves; HP2, the second day of the high-protein diet for calves; LP1, the first day of the low-protein diet for calves; LP2, the second day of the low protein diet for calves; SEM, standard error of mean.

**Table 3:** Alpha-diversity indices of microbiota in the rumen of calves after the 4 days of one oscillation period.

Items	HP1	HP2	LP1	LP2	SEM	*p* Value
Chao1	999.8	1004.4	1008.2	1003.4	3.33	0.867
Ace	989.2	999.3	1002.8	994.4	2.97	0.421
Shannon	5.2	5.2	5.4	5.3	0.04	0.167
Simpson	0.025	0.027	0.018	0.026	0.0017	0.196
Coverage	>99%	>99%	>99%	>99%		

*n* = 6 for each treatment. *p* value < 0.05 was defined as statistically significant. HP1, the first day of high-protein diet for calves; HP2, the second day of high-protein diet for calves; LP1, the first day of low-protein diet for calves; LP2, the second day of low-protein diet for calves; SEM, standard error of mean.

## Data Availability

16S rRNA sequence data sets are publicly available through NCBI’s Sequence Read Archive, under accession number PRJNA722793.
